# A Case of Sarcoidosis Associated With Anti–Tumor Necrosis Factor Treatment

**DOI:** 10.1177/2324709615571366

**Published:** 2015-03-09

**Authors:** Ayse Baha, Cigdem Hanazay, Nurdan Kokturk, Haluk Turktas

**Affiliations:** 1 Gazi University School of Medicine, Department of Pulmonary Medicine, Ankara, Turkey

**Keywords:** pulmonary sarcoidosis, anti-TNF-α treatment, psoriasis

## Abstract

Sarcoidosis is a systemic chronic granulomatous disease of unknown etiology. It predominantly involves the lungs but can affect many organs or tissues in the body, such as the lymphatic system, skin, eyes, and liver. Typical histopathological lesions are noncaseating granulomas in the affected organ or tissue. Indications, type of treatment, and duration of sarcoidosis treatment is currently debated. Despite studies showing that anti–tumor necrosis factor-α (TNF-α) treatment can successfully be used in refractory sarcoidosis, there are some case reports regarding the development of sarcoidosis with these agents. There have been reports of 47 anti-TNF-associated cases of sarcoidosis until 2012. The patient is a 54-year-old Caucasian male. During routine examinations of the patient who had been followed for psoriasis vulgaris for 20 years and who had been on several anti-TNF regimens thereafter, new pulmonary pathologies due to sarcoidosis were detected. We present here a case of sarcoidosis that developed after infliximab treatment and showed obvious radiologic regression with discontinuation of treatment. During anti-TNF treatment, it should be kept in mind that autoimmune and granulomatous diseases may develop and particular care should be given to patient follow-ups.

## Introduction

Sarcoidosis is a multisystem granulomatous disease that generally involves lungs and intrathoracic lymph nodes, and it has an unknown etiology. While it is more common in adults, children can also be affected. It is most frequently observed in adults in their 30s and peaks in their 50s. Disease involvement and clinical course differ by ethnicity. Its etiology is unknown; however, genetic transition is thought to be influential. Although debates regarding the treatment still continue, there is some information about sarcoidosis development, thanks to anti–tumor necrosis factor (TNF) agents used in steroid-resistant sarcoidosis and relapses during systematic involvement. There have been reports of 47 anti-TNF-associated cases of sarcoidosis until 2012.

## Case

**Figure 1.**
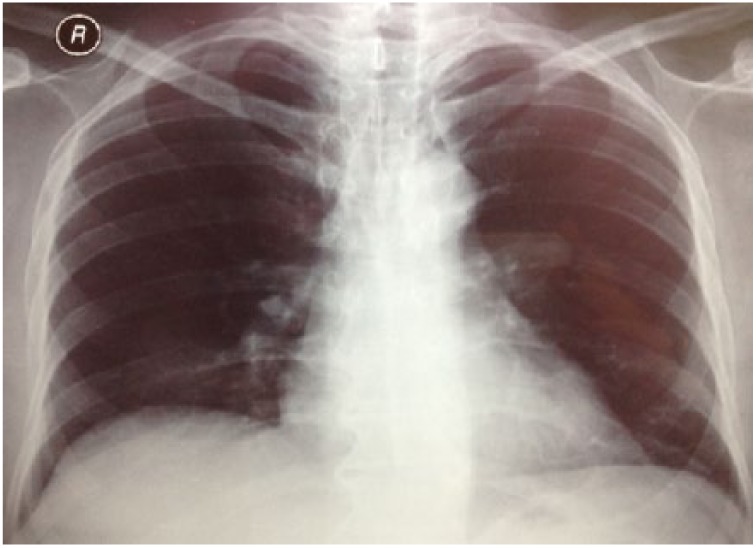
The control chest x-ray before anti-TNF treatment (March 2010).

**Figure 2.**
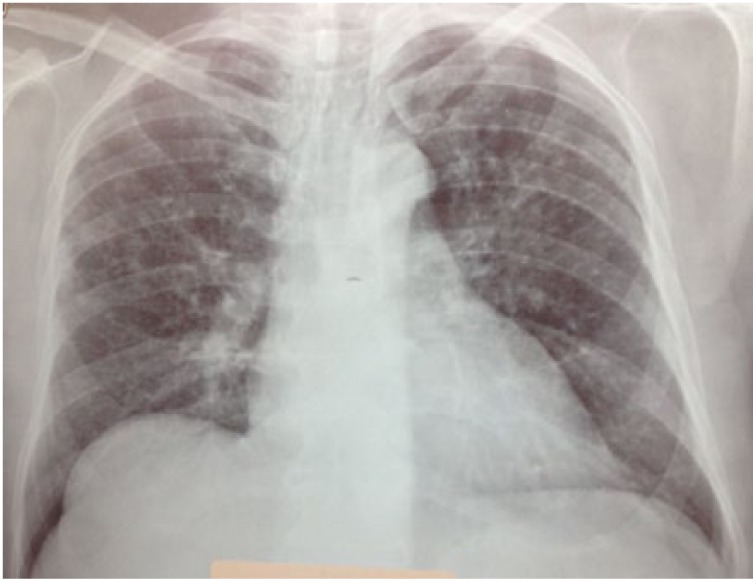
The chest x-ray during anti-TNF treatment (August 2012).

**Figure 3.**
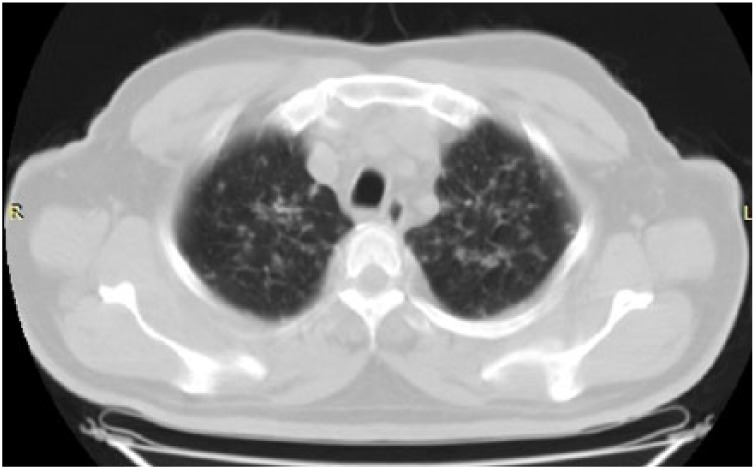
Bilateral millimetric nodules and interstitial thickening in peribrochovascular areas of upper zones are shown.

**Figure 4.**
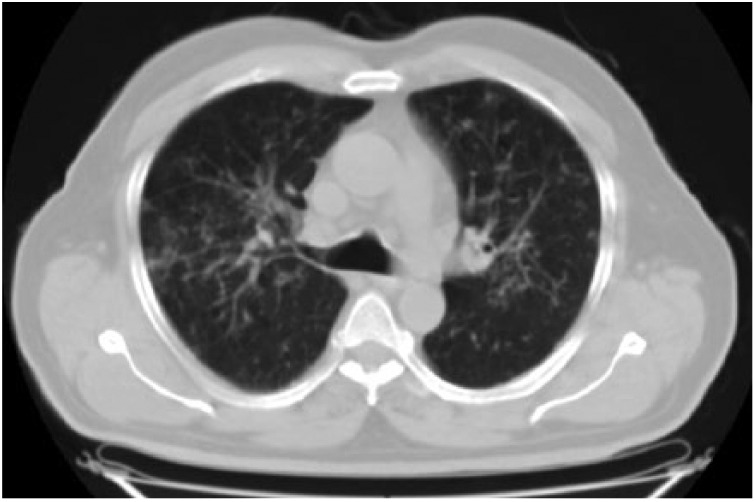
Parenchymal window showing widespread millimetric nodules and interlobular thickening in peribrochovascular and subpleural areas of bilateral upper zones.

**Figure 5.**
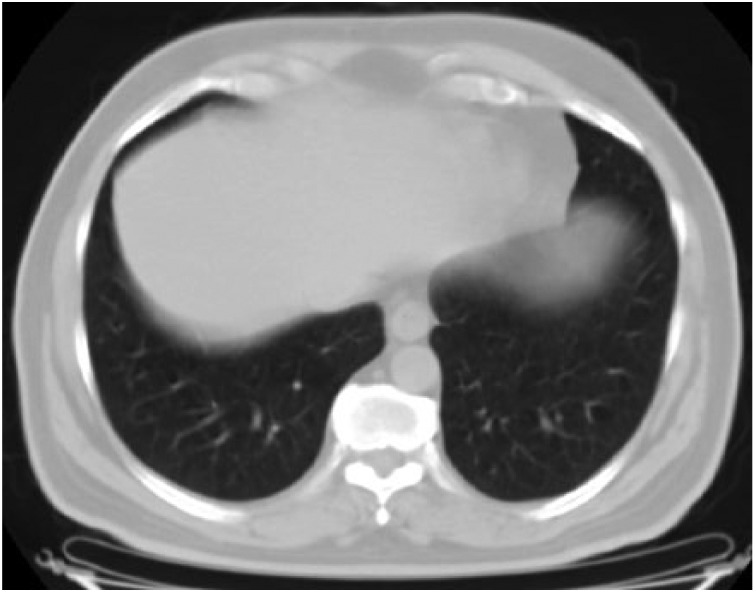
Parenchymal window showing unaffected areas in bilateral lower lobes.

**Figure 6.**
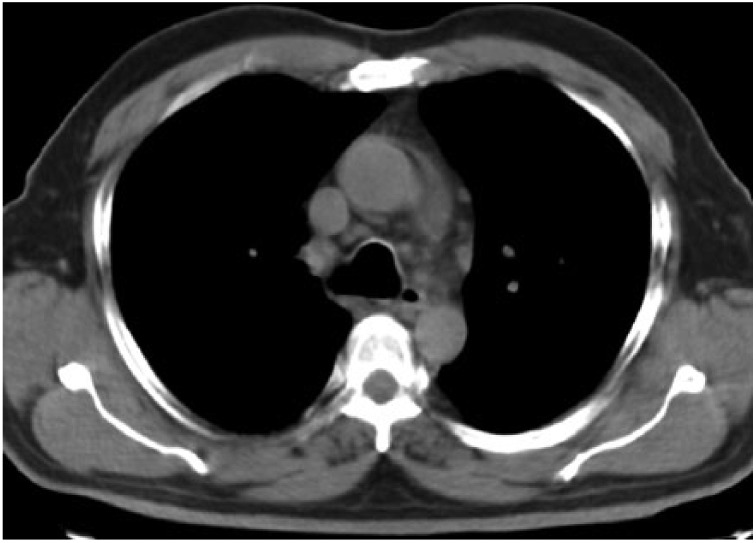
Mediastinal window showing lymph nodes in stations 4R, 5, and 6.

**Figure 7.**
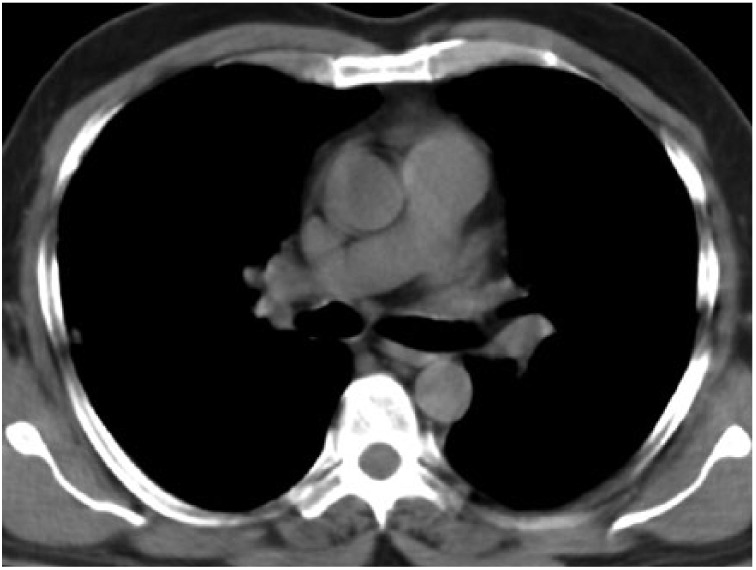
Mediastinal window showing lymph nodes in stations 10R and 10L.

A 54-year-old male patient was diagnosed with psoriasis vulgaris 20 years ago. He received methotrexate treatment for 6 months in 2005. However, the treatment was discontinued due to elevated liver function tests. Following exacerbation of his skin lesions, he received cyclosporine treatment for 4 months in February 2008. Again, the treatment was discontinued due to elevated liver function tests and severe enteritis. After 3 months, he received etanercept (ETN) treatment due to exacerbation of his disease. The ETN treatment was discontinued after 59 cycles due to elevated carbohydrate antigen 199 levels in the follow-up examinations. The patient was scheduled for infliximab (IFX) therapy due to increased symptoms in March 2010. There was no sign of infiltration in the chest x-ray (Figure 1), and the lung examination was normal. Besides, there were no symptoms regarding organs except for the skin, and abdominal ultrasonography and colonoscopic evaluations were normal. The patient’s anti-HBs, anti-HCV, and anti-HIV antibodies were negative, while C-reactive protein, procalcitonin, whole blood count, and liver and kidney function tests were at the normal levels. The patient was started on anti-TNF treatment. The patient received isoniazid prophylaxis for 9 months (1 × 300 mg/day) after a tuberculin skin test (ppd = 16 mm), and chest x-ray and physical examinations were normal until August 2012. At that date, bilateral hilar swelling and a reticulonodular appearance at the bilateral middle and lower zones were detected during bilateral chest x-ray (Figure 2). The patient was prediagnosed with sarcoidosis and tuberculosis, and a high-resolution thorax computed tomography (CT) was planned. The following parameters were detected in the 24-hour urine sample: calcium, 383 mg/24 h (100-300 mg/24 h); serum calcium, 9.5 mg/dL (8.2-10.6 mg/dL), angiotensin-converting enzyme level was high, and ppd was 15 mm. There were no significant signs of sarcoidosis and tuberculosis involvement in the eyes. The following results were found in diffusion respiratory function test: forced expiratory volume in 1 second (FEV_1_) 2860 cc 87%; forced vital capacity (FVC) 3370 cc 83%; FEV_1_/FVC 85%; diffusing capacity for carbon monoxide 77%; DLCO/alveolar volume (VA) 112; RV (residual volume) 130%; TLC (total lung capacity) 99%; and VC (vital capacity) 85%. High-resolution CT revealed peribronchovascular nodules in the bilateral upper and middle zones that were centrally located (Figures 3-5); hilar and mediastinal lymphadenopathies were also detected (Figures 6 and 7).

The patient underwent fiberoptic bronchoscopy. Bronchoalveolar lavage (BAL) was obtained from the anterior segment of the upper left lung. Mucosal biopsy specimens were obtained from the upper left lung and tracheal carinas of the upper left lung. Transbronchial biopsy (TBB) specimen were obtained from the anterior segment of the upper left lung. Fifty-five percent lymphocytes, 40% macrophages, and 5% neutrophils were detected in the BAL specimen, and non-necrotizing granulomatous lesions were detected in the TBB specimens. During histopathological examination of the biopsy specimens, Ehrlich-Ziehl-Neelsen and Kinyoun-specific microorganisms were not detected. The tuberculosis culture from the tissue biopsy was negative. The examination of histopathological and microbiological specimens did not yield any fungal elements or microorganisms including histoplasmosis. The BAL panfungal polymerase chain reaction was negative for the fungi. As no cases of histoplasmosis have been reported in our center, we did not specifically study histoplasmosis.

**Figure 8.**
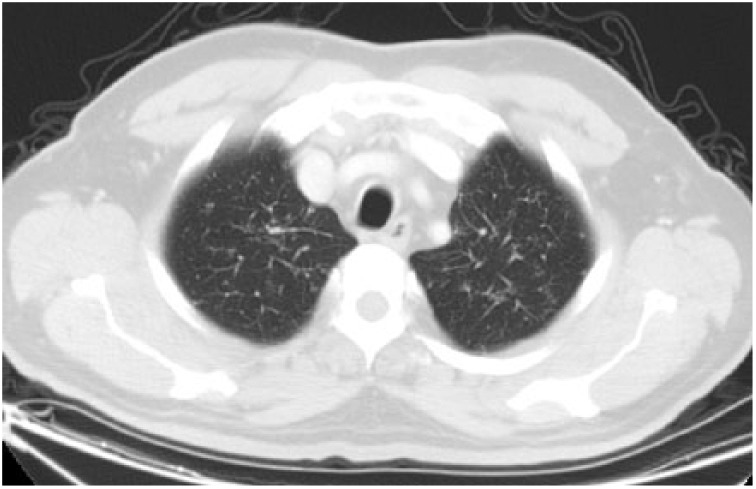
Control thorax HRCT parenchymal window showing regression of nodules and interstitial thickening in bilateral upper zones.

**Figure 9.**
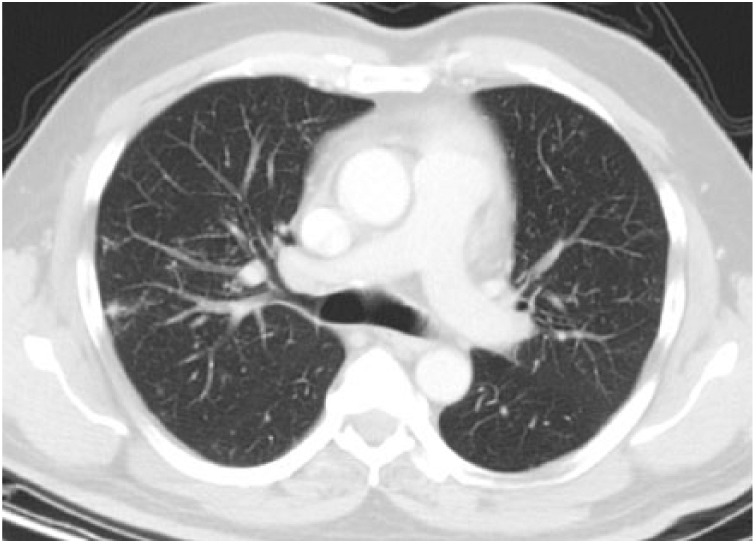
Control thorax HRCT parenchymal window showing regression of nodules and interstitial shadows.

**Figure 10.**
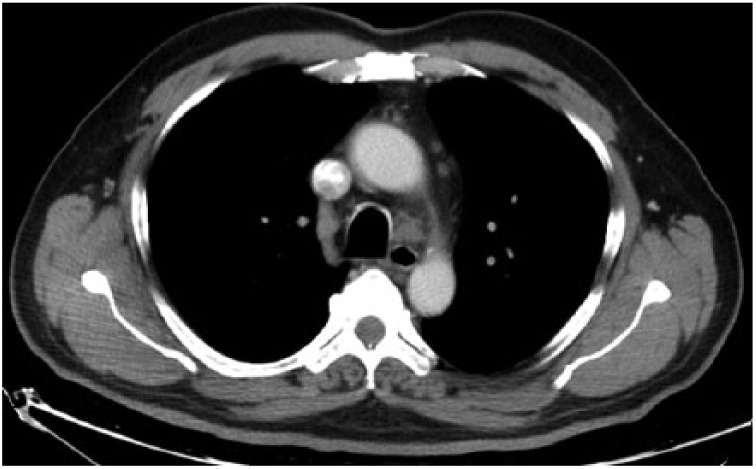
Control thorax HRCT with mediastinal window showing regression in the size of mediastinal lymph nodes.

**Figure 11.**
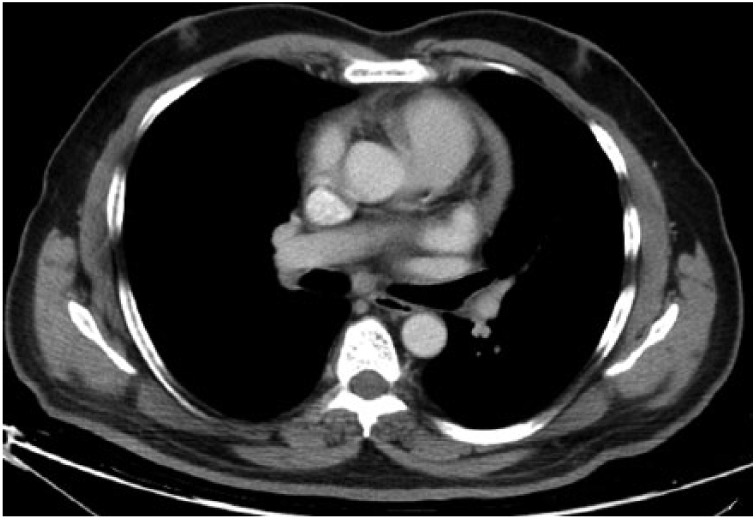
Control thorax HRCT with mediastinal window showing disappearance of hilar lymph nodes.

Considering the radiological and pathological findings, the patient was diagnosed with stage 2 sarcoidosis and was suggested to discontinue anti-TNF treatment. After discontinuation, the patient had been followed for 4 months without any treatment and showed no symptoms. Thereafter, we observed regression in the control chest x-ray and high-resolution CT (Figures 8-11), and the patient is now on topical treatment for psoriasis without any treatment for sarcoidosis.

## Discussion

Anti-TNF-α agents are used to treat various chronic inflammatory diseases (rheumatoid arthritis, spondyloarthropathies, psoriasis, and Crohn’s disease). In recent years, there have been many publications indicating that various autoimmune diseases and interstitial lung diseases develop or preexisting lung diseases deteriorate depending on these agents.^1,2^ Sarcoidosis, with unknown etiology, is a disease characterized by non-necrotizing granulomas and might affect all the organs and systems, particularly adenoids and the lungs. Regarding sarcoidosis treatment, the indications, type of treatment, and duration are currently debated. However, according to case reports anti-TNF agents, which are among the possible options for refractory sarcoidosis treatment, cause granulomatous lesions.^3,4^

A possible hypothesis to explain the pathogenesis of anti-TNF-induced granulomatous disease could rise from another paradoxical phenomenon linked to these drugs, that is, the psoriatic lesions that develop during the course of anti-TNF therapy.^5^ The paradoxical aspect of this phenomenon is that TNF is one of the molecules involved in the induction of psoriatic skin lesions, and all TNF antagonists have repeatedly been shown to be efficacious in both cutaneous and articular disease. The appearance of psoriasis with all 3 anti-TNF agents is considered to be a “class-dependent” effect rather than an immuno-allergic reaction.^6^ Therefore, it is possible that both phenomena could be viewed in the context of a cytokine imbalance due to prolonged TNF suppression. Indeed, the occurrence of autoimmune diseases related to anti-TNF agents, such as “lupus-like” syndrome and vasculitis, is well known with such therapy.^7^

Here, we present a case of pulmonary sarcoidosis that developed during anti-TNF-α treatment for psoriasis and showed spontaneous regression after discontinuation of anti-TNF treatment.

The study by Ramos-Casals et al, which is regarded as one of the most comprehensive studies on the anti-TNF-α-associated sarcoidosis cases, evaluated 10 cases with sarcoidosis. Eight of the cases developed sarcoidosis during ETN treatment, while 2 cases developed sarcoidosis during IFX treatment. Treatment of ILD involved the withdrawal of anti-TNF therapy in 8 cases, and corticosteroids were used in 5 patients. Resolution was observed in 7 patients, improvement in 2 patients, and there was no resolution in 1 patients; no deaths were reported.^8^

According to a literature search by Tong et al, 37 cases of anti-TNF treatment-associated sarcoidosis were determined between 2000 and 2009, and the authors reported 3 additional cases that were diagnosed by themselves.^9^

There have been reports of 47 anti-TNF-associated cases of sarcoidosis until 2012.^10^ Nine of these cases received IFX, 8 cases received adalimumab, and 30 cases received ETN. In 5 of the cases that developed sarcoidosis during IFX treatment, clinical and radiological resolution (full resolution) was achieved, whereas only clinical resolution was achieved in 1 case. In 2 out of 9 cases, additional systemic steroid treatment (to discontinued IFX treatment) led to complete resolution, while clinical resolution was observed in 1 case. In our case, discontinuation of the IFX treatment led to almost complete clinical and radiological resolution during the 4-month follow-up period.

During anti-TNF treatment, various infectious complications, particularly tuberculosis, may occur.^11^ In our case, we excluded the possible tuberculosis diagnosis as there were no pulmonary symptoms and tuberculosis bacilli were not detected in the BAL and lung tissue biopsy cultures. Considering the current laboratory findings and clinical, radiological, and pathological features, the case was regarded as sarcoidosis and determined to have resolution after IFX treatment was discontinued. During anti-TNF treatment, it should be kept in mind that autoimmune and granulomatous diseases may develop and particular care should be given to patient follow-ups.
